# Redetermination of 2,4,6-tricyclo­hexyl-1,3,5-trioxane

**DOI:** 10.1107/S1600536808018084

**Published:** 2008-06-19

**Authors:** Rodolfo Moreno-Fuquen, Eunice Rios, Rodrigo Paredes, Luz Marina Jaramillo, Julio Zukerman-Schpector

**Affiliations:** a Departamento de Química, Facultad de Ciencias, Universidad del Valle, Apartado 25360, Santiago de Cali, Colombia; b Departamento de Química, Facultad de Ciencias, Universidad del Quindio, Armenia, Colombia; c Departamento de Química, Facultad de Ciencias, Universidad del Valle, Apartado 25360, Santiago de Cali, Colombia; d Departmento de Química, Universidade Federal de São Carlos, São Carlos, SP, Brazil

## Abstract

The title compound, C_21_H_36_O_3_, was obtained by treatment of cyclo­hexa­necarbaldehyde with catalytic toluene-4-sulfonic acid monohydrate. This redetermination results in a crystal structure with significantly higher precision than the original determination [Diana & Ganis (1963 ▶). *Atti Accad. Naz. Lincei*, **35**, 80–88]. The asymmetric unit contains one sixth of the mol­ecule, the formula unit being generated by crystallographic 3*m* symmetry. In the mol­ecule, the trioxane and cyclo­hexane rings are in chair conformations. In the crystal structure, mol­ecules are linked by weak C—H⋯O hydrogen bonds along the [001] direction.

## Related literature

For related literature, see: Augé & Gil (2002 ▶); Etter (1990 ▶); Ho & Lee (2001 ▶); Iulek & Zukerman-Schpector (1997 ▶); Johnson *et al.* (1996 ▶); Nardelli (1995 ▶); Diana & Ganis (1963 ▶).
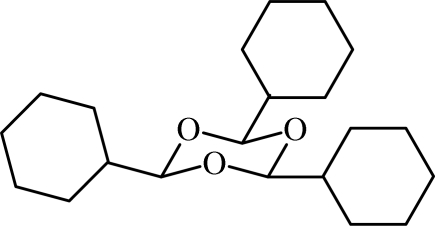


## Experimental

### 

#### Crystal data

C_21_H_36_O_3_
*M* *_r_* = 336.50
Hexagonal, 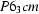

*a* = 11.8542 (3) Å
*c* = 7.9908 (3) Å
*V* = 972.44 (5) Å^3^
*Z* = 2
Mo *K*α radiation
μ = 0.07 mm^−1^
*T* = 298 K
0.21 × 0.18 × 0.08 mm

#### Data collection

Enraf–Nonius CAD-4 diffractometer
Absorption correction: none
1372 measured reflections
439 independent reflections
382 reflections with *I* > 2σ(*I*)
*R*_int_ = 0.026
2 standard reflections frequency: 150 min intensity decay: 0.1%

#### Refinement

*R*[*F* ^2^ > 2σ(*F* ^2^)] = 0.035
*wR*(*F* ^2^) = 0.096
*S* = 1.18
439 reflections
43 parameters
1 restraint
H-atom parameters constrained
Δρ_max_ = 0.17 e Å^−3^
Δρ_min_ = −0.17 e Å^−3^

### 

Data collection: *CAD-4 Software* (Enraf–Nonius, 1989 ▶); cell refinement: *CAD-4 Software*; data reduction: *CAD-4 SDP* (Frenz, 1978 ▶); program(s) used to solve structure: *SHELXS97* (Sheldrick, 2008 ▶); program(s) used to refine structure: *SHELXL97* (Sheldrick, 2008 ▶); molecular graphics: *ORTEP-3 for Windows* (Farrugia, 1997 ▶); software used to prepare material for publication: *PARST95* (Nardelli, 1995 ▶).

## Supplementary Material

Crystal structure: contains datablocks I, global. DOI: 10.1107/S1600536808018084/lh2635sup1.cif

Structure factors: contains datablocks I. DOI: 10.1107/S1600536808018084/lh2635Isup2.hkl

Additional supplementary materials:  crystallographic information; 3D view; checkCIF report

## Figures and Tables

**Table 1:** Hydrogen-bond geometry (Å, °)

*D*—H⋯*A*	*D*—H	H⋯*A*	*D*⋯*A*	*D*—H⋯*A*
C1—H1⋯O1^i^	0.98	2.56	3.534 (3)	176

Symmetry code: (i) 
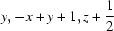
.

**Fig. 1.**
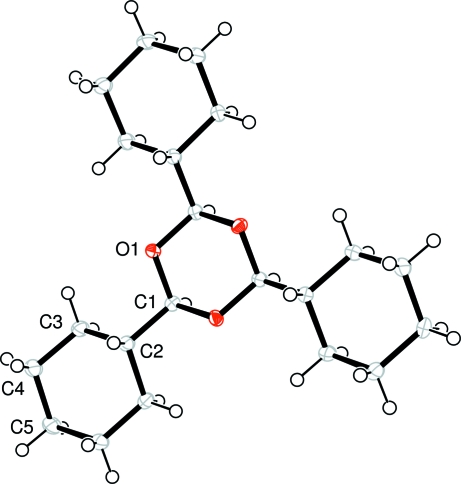
An ORTEP-3 (Farrugia, 1997) plot of the (I) compound, with the atomic labelling scheme (for the asymmetric unit). The shapes of the ellipsoids correspond to 50% probability contours of atomic displacement and, for the sake of clarity, H atoms are shown as spheres of arbitrary radius.

**Fig. 2.**
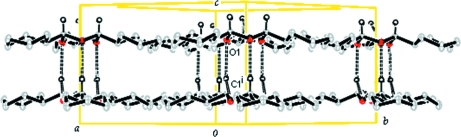
View normal to (001) of the crystal structure of (I). [Symmetry code: (i) -x+y+1, y, z-1/2]. Weak C—H..O hydrogen bonds are shown as dashed lines.

**Fig. 3.**
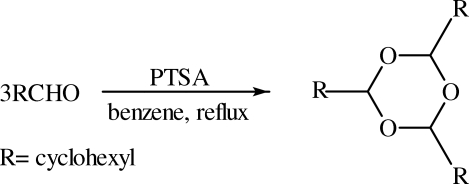
Reaction scheme

## supplementary crystallographic information

### Comment

Trioxanes have many applications in different fields such as insecticides, flavouring materials and stabilizers in colour photography (Augé, & Gil, 2002). Several methods have been reported for the synthesis of 1,3,5-trioxanes from aldehydes (Johnson *et al.*, 1996). The synthesis of a wide variety of 1,3,5-trioxanes using acetonyltriphenylphosphonium bromide as catalyst are reported (Ho & Lee, 2001). In a new efficient method, using trimethylsilyl chloride as a catalyst of aldehydes, 1,3,5 trioxanes were formed (Augé & Gil, 2002). As an alternative way of obtaining trioxane compounds, the use in the reaction of toluene-4-sulfonic acid monohydrate (PTSA) as a catalizator, is proposed in the present work. The title compound, C_21_H_36_O_3_, 2,4,6-trialkyl-1,3,5-trioxane, (I) was obtained by treatment of cyclohexanecarbaldehyde with catalytic PTSA (Fig. 3). The molecular structure of (I), showing the atomic numbering scheme, can be seen in Fig. 1. The crystal structure of (I) is stabilized by weak intermolecular C—H···O hydrogen-bonds (Nardelli, 1995) (Table 1). The atom C1 in the molecule at (*x*, *y*, *z*) acts as a hydrogen-bond donor to atom O1 in the molecule at (*y*, -*x* + *y*+1, 1/2 + *z*), so forming C(3) chains (Etter, 1990) along [001] direction (Fig. 2). The conformation of trioxane ring is of the pure chair, as indicated by the Cremer & Pople puckering parameters (Iulek & Zukerman-Schpector, 1997), being q_2_ = 0.00 Å, q_3_ = -0.565 Å, φ_2_ = 0°, τ = 180°, and a puckering amplitude of Q_T_ = 0.565 Å and the conformation of the cyclohexane ring is of the chair and its puckering parameters are: q_2_ = 0.0359 Å, q_3_ = -0.5743 Å, φ_2_ = 180°, τ = 176.4°, and a puckering amplitude of Q_T_ = 0.575 Å.

### Experimental

The title compound was prepared by adding 2.0 g of cyclohexanecarbaldehyde (17.8 mmol) to benzene (20 ml). To this solution 0.200 g of PTSA.H_2_O (1.05 mmol) was added. The mixture was refluxed for 6 h and then it was cooled overnight in the refrigerator. The solid formed, a trimeric complex, was separated and dried. 0.20 g of PTSA.H_2_O (1.05 mmol) was added to an acetone–water (3:1) solution (20 ml). To this solution 0.500 g of trimeric complex (2.23 mmol) was added. The mixture was stirred for 5 minutes and then the solid was filtered and dried. The product was recrystalized from ethyl ether. This last compound was identified as (I) on the basis of its spectra and X-ray analysis. *cis*,*cis*-2,4,6-tricyclohexyl-1,3,5-trioxane. Colourless crystals; yield 76%; mp 435 (1) K. IR (KBr) 2923, 2851, 1161, 1124, 1068 cm^-1^; δ_H_ (300 MHz; CDCl_3_; Me_4_Si) 0.99–1.21 (15H, m, equatorial Hs in cyclohexyl groups), 1.56–183 (18H, m, axial Hs in cyclohexyl groups), and 4.47 (3H, d) [lit., 1.01–1.24 (15H, m), 1.58–1.83 (18H, m) and 4.49 (3H, d)]; δ_C_ (75 MHz; CDCl_3_; Me_4_Si) 25.655, 26.466, 27.038, 41.865 and 104.292 (lit., 25.6, 26.5, 27.0, 41.9 and 104.3); m/*z*(EI) 336 (*M*^+^, 2%), 95 (100).

Crystals for X-ray diffraction were grown from a solution of the title compound in diethyl ether.

### Refinement

In the absence of significant anomalous dispersion effects the Friedel pairs were merged before refinement. All H-atoms were located in difference maps and then treated as riding atoms [C—H = 0.93 Å and *U*_iso_(H) = 1.2*U*_eq_(C)].

### Crystal data

**Table:**

C_21_H_36_O_3_	*Z* = 2
*M**_r_* = 336.50	*F*_000_ = 372
Hexagonal, *P*6_3_*c**m*	*D*_x_ = 1.149 Mg m^−^^3^
Hall symbol: P 6c -2	Melting point: 435(1) K
*a* = 11.8542 (3) Å	Mo *K*α radiation λ = 0.71073 Å
*b* = 11.8542 (3) Å	Cell parameters from 25 reflections
*c* = 7.9908 (3) Å	θ = 3.0–25.0º
α = 90º	µ = 0.07 mm^−^^1^
β = 90º	*T* = 298 K
γ = 120º	Plate, colourless
*V* = 972.44 (5) Å^3^	0.21 × 0.18 × 0.08 mm

### Data collection

**Table:**

Enraf–Nonius CAD-4 diffractometer	θ_max_ = 27.5º
Radiation source: fine-focus sealed tube	θ_min_ = 3.4º
Monochromator: graphite	*h* = 1→15
ω/2θ scans	*k* = −15→0
Absorption correction: none	*l* = −10→10
1372 measured reflections	2 standard reflections
439 independent reflections	every 150 min
382 reflections with *I* > 2σ(*I*)	intensity decay: 0.1%
*R*_int_ = 0.026	

### Refinement

**Table:**

Refinement on *F*^2^	Secondary atom site location: difference Fourier map
Least-squares matrix: full	Hydrogen site location: inferred from neighbouring sites
*R*[*F*^2^ > 2σ(*F*^2^)] = 0.035	H-atom parameters constrained
*wR*(*F*^2^) = 0.096	*w* = 1/[σ^2^(*F*_o_^2^) + (0.0528*P*)^2^ + 0.1112*P*] where *P* = (*F*_o_^2^ + 2*F*_c_^2^)/3
*S* = 1.18	(Δ/σ)_max_ < 0.001
439 reflections	Δρ_max_ = 0.17 e Å^−^^3^
43 parameters	Δρ_min_ = −0.17 e Å^−^^3^
1 restraint	Extinction correction: none
Primary atom site location: structure-invariant direct methods	

### Special details

**Table:**

Geometry. All e.s.d.'s (except the e.s.d. in the dihedral angle between two l.s. planes) are estimated using the full covariance matrix. The cell e.s.d.'s are taken into account individually in the estimation of e.s.d.'s in distances, angles and torsion angles; correlations between e.s.d.'s in cell parameters are only used when they are defined by crystal symmetry. An approximate (isotropic) treatment of cell e.s.d.'s is used for estimating e.s.d.'s involving l.s. planes.

### Fractional atomic coordinates and isotropic or equivalent isotropic displacement parameters (Å^2^)

**Table:**

	*x*	*y*	*z*	*U*_iso_*/*U*_eq_	
O1	1.0000	0.88684 (11)	0.6920 (2)	0.0148 (4)	
C1	0.88552 (16)	0.88552 (16)	0.7497 (3)	0.0145 (6)	
H1	0.8819	0.8819	0.8722	0.017*	
C2	0.76794 (17)	0.76794 (17)	0.6771 (3)	0.0162 (5)	
H2	0.7743	0.7743	0.5549	0.019*	
C3	0.76350 (16)	0.64144 (15)	0.7298 (2)	0.0208 (5)	
H31	0.8430	0.6440	0.6945	0.025*	
H32	0.7582	0.6339	0.8508	0.025*	
C4	0.64605 (15)	0.52264 (14)	0.6519 (3)	0.0257 (5)	
H41	0.6559	0.5258	0.5312	0.031*	
H42	0.6430	0.4438	0.6917	0.031*	
C5	0.51833 (18)	0.51833 (18)	0.6966 (3)	0.0233 (6)	
H51	0.5020	0.5020	0.8155	0.028*	
H52	0.4471	0.4471	0.6369	0.028*	

### Atomic displacement parameters (Å^2^)

**Table:**

	*U*^11^	*U*^22^	*U*^33^	*U*^12^	*U*^13^	*U*^23^
O1	0.0117 (7)	0.0142 (6)	0.0176 (11)	0.0058 (4)	0.000	−0.0021 (5)
C1	0.0153 (9)	0.0153 (9)	0.0142 (14)	0.0087 (8)	0.0007 (7)	0.0007 (7)
C2	0.0147 (8)	0.0147 (8)	0.0189 (14)	0.0072 (8)	0.0010 (8)	0.0010 (8)
C3	0.0171 (7)	0.0166 (8)	0.0300 (13)	0.0095 (6)	−0.0013 (7)	0.0025 (7)
C4	0.0186 (8)	0.0143 (7)	0.0428 (14)	0.0072 (6)	−0.0014 (8)	−0.0001 (8)
C5	0.0164 (8)	0.0164 (8)	0.0315 (17)	0.0041 (9)	0.0041 (9)	0.0041 (9)

### Geometric parameters (Å, °)

**Table:**

O1—C1	1.426 (3)	C3—H32	0.9700
C1—C2	1.509 (3)	C4—C5	1.531 (2)
C1—H1	0.9800	C4—H41	0.9700
C2—C3	1.5328 (18)	C4—H42	0.9700
C2—H2	0.9800	C5—H51	0.9700
C3—C4	1.532 (2)	C5—H52	0.9700
C3—H31	0.9700		
			
C1^i^—O1—C1	111.0 (2)	C4—C3—H32	109.4
O1^ii^—C1—O1	109.12 (19)	C2—C3—H32	109.4
O1^ii^—C1—C2	108.68 (14)	H31—C3—H32	108.0
O1—C1—C2	108.68 (13)	C5—C4—C3	111.40 (16)
O1^ii^—C1—H1	110.1	C5—C4—H41	109.3
O1—C1—H1	110.1	C3—C4—H41	109.3
C2—C1—H1	110.1	C5—C4—H42	109.3
C1—C2—C3	111.23 (12)	C3—C4—H42	109.3
C1—C2—H2	108.2	H41—C4—H42	108.0
C3—C2—H2	108.2	C4—C5—H51	109.3
C4—C3—C2	111.01 (14)	C4—C5—H52	109.3
C4—C3—H31	109.4	H51—C5—H52	108.0
C2—C3—H31	109.4		
			
C1^i^—O1—C1—O1^ii^	58.5 (3)	O1—C1—C2—C3	59.4 (2)
C1^i^—O1—C1—C2	176.87 (11)	C1—C2—C3—C4	−178.44 (18)
O1^ii^—C1—C2—C3	178.04 (17)	C2—C3—C4—C5	−56.1 (2)

Symmetry codes: (i) −*y*+2, *x*−*y*+1, *z*; (ii) −*x*+*y*+1, −*x*+2, *z*.

### Hydrogen-bond geometry (Å, °)

**Table:**

*D*—H···*A*	*D*—H	H···*A*	*D*···*A*	*D*—H···*A*
C1—H1···O1^iii^	0.98	2.56	3.534 (3)	176

Symmetry codes: (iii) *y*, −*x*+*y*+1, *z*+1/2.
